# Gut microbiota changes in animal models of spinal cord injury: a preclinical systematic review and meta-analysis

**DOI:** 10.1080/07853890.2023.2269379

**Published:** 2023-10-18

**Authors:** Zhenye Zhang, Nan Cheng, Jianfen Liang, Yifan Deng, Ping Xiang, Ziqing Hei, Xiang Li

**Affiliations:** a Department of Anesthesiology, The Third Affiliated Hospital, Sun Yat-Sen University, Guangzhou, China; b Department of Medical Quality Management, Nanfang Hospital, Southern Medical University, Guangzhou, China

**Keywords:** Gut microbiota, spinal cord injury, meta analysis

## Abstract

**Background:**

An increasing number of studies show that the intestinal flora is closely related to spinal cord injury. Many researchers are exploring the changes in the richness, diversity, and evenness of intestinal flora in spinal cord injury animal models to identify the characteristic bacteria.

**Methods:**

A comprehensive literature search was conducted using three databases: PubMed, Embase, and Web of Science. A meta-analysis was performed using R 4.3.1 to evaluate the comparison of microbiota diversity, richness, and evenness and the relative abundance of intestinal microbiota in animals with spinal cord injury and blank controls.

**Results:**

Fifteen studies were included in the meta-analysis, of which 12 involved gut microbiota distribution indicators and 11 included intestinal microflora relative abundance indicators. Meta-analysis of high-dimensional indicators describing the distribution of the gut microbiota identified a substantial decline in the evenness and richness of the intestinal flora. In addition, the Actinobacteria phylum and Erysipelotrichales and Clostridiales orders were significantly different between the spinal cord injury and sham groups; therefore, they may be the characteristic bacteria in spinal cord injury models.

**Conclusion:**

Our meta-analysis suggested that the gut microbiota in the spinal cord injury animal model group was altered compared with that in the control group, with varying degrees of changes in richness and evenness and potentially pathogenic characteristic flora. More rigorous methodological studies are needed because of the high heterogeneity and limited sample size. Further research is needed to clinically apply intestinal microbiota and potentially guide fecal microbiota transplantation therapy.

## Key Messages

Our meta-analysis showed that spinal cord injury significantly decreased the richness and evenness of intestinal flora in experimental animals.

No statistically significant changes in the phyla flora during spinal cord injury have been found.

Erysipelotrichales and Clostridiales may be the characteristic flora of gut microbiota changes during spinal cord injury.

## 1. Introduction

Spinal cord injury (SCI) is a common and severe neurological disease usually caused by acute external mechanical injury [1], including traffic accidents and serious falls [2]. Epidemiological studies show that SCI prevalence is the highest in the United States of America at 250 per million cases every year [3]. SCI can seriously damage the physical and mental health of patients, affect social development, and impose a huge economic burden on the healthcare system; therefore, this disease cannot be ignored [4].

SCIs can be devastating for several reasons based on the internal metabolic, biological, and pathophysiological changes that occur [5]. Disorder of the spinal cord environment after SCI is an important reason for the death of neurons in the injured area and difficult recovery of motor function [6]. Secondary pathophysiological changes after SCI (such as inflammation, edema, metabolic disorders, and other complications) further aggravate the injury [7]. Researchers are beginning to see the significant impact of characteristic microbiota on certain diseases. This provides new ideas for treatment. Fecal microbiota transplantation (FMT) stands out and has already shown some success in gastrointestinal diseases and neuropsychiatric disorders [8,9]. Therefore, FMT can be regarded as a potential treatment for complex systemic reactions after SCI to help the body cope with the adverse effects of injury such as changes in inflammatory factors and hyperalgesia. Many researchers are exploring the efficacy of FMT for SCI [10]. Changes in the diversity and richness of gut microbiota and relative abundance after SCI affect the original function of intestinal flora in regulating inflammatory response and metabolism and affect SCI progression and its complications *via* changes in the neuroendocrine system and intestinal brain axis [6]. The gut microbiome regulates various cellular and molecular mechanisms that are critical for the progression of pathophysiological processes induced by acute central nervous system damage [11,12]. Changes in the gut flora can disrupt the balance of the bidirectional enterobrain axis. This exacerbates secondary brain injury, and impairs cognitive function and motor function that leads to a poor prognosis by triggering pro-inflammatory responses in the peripheral circulation and central nervous system [11]. For example, diseases disrupt the balance of the gut-brain axis by affecting the absorption of food-related metabolites. Short-chain fatty acids (SCFAs) act on various targets by activating free fatty acid 2 receptors and are involved in everything from neuroplasticity to gene expression, food intake, and immune system regulation. Patients with irritable bowel syndrome lack SCFA-producing flora. The application of SCFA-producing gut microbiota in germ-free mice inhibits this activation; this suggests that a healthy gut microbiome can inhibit this neuronal signaling pathway [13,14]. The intestine is innervated by the parasympathetic vagus and sympathetic spinal nerves in SCIs that originate in the brainstem and spinal cord. Damaged nociceptors dominate the intestine and release inflammatory mediators following SCI. This can lead to a neurogenic bowel with patients experiencing constipation, fecal incontinence, and bloating [9,15,16].

Intestinal flora animal models are being used to determine the cause of aggravated SCI [17]. In addition, some researchers conclude that FMT plays a neuroprotective role by regulating the microenvironment of the lesion site in a mouse model of SCI [18].

To date, several systematic reviews show associations between SCI and the gut microbiome. However, meta-analyses were not performed. This systematic review and meta-analysis investigated the association between SCI and intestinal flora in animal models and explored the characteristic changes.

## 2. Methods

### 2.1. Search strategy

This systematic review and meta-analysis followed the Preferred Reporting Items for Systematic Reviews and Meta-Analyses (PRISMA) guidelines [19].

**Figure 1.**
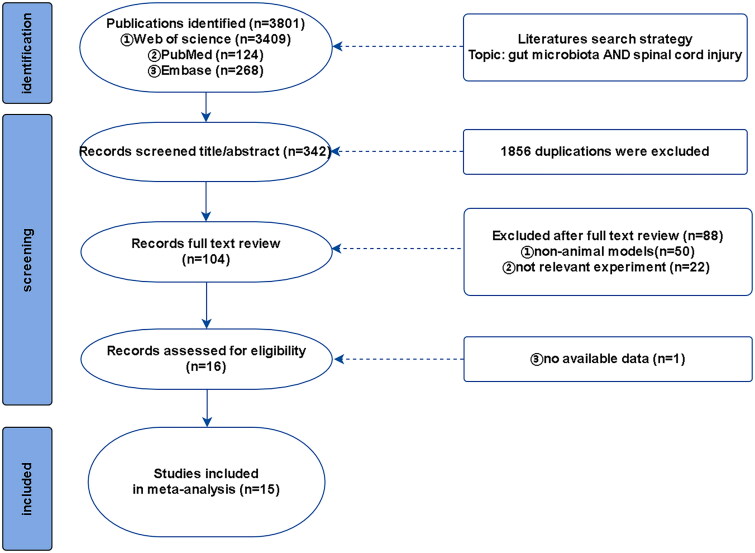
Flow diagram of literature search.

A comprehensive search was conducted in PubMed, Embase, and Web of Science using keywords such as ‘spinal cord injury’ and ‘gut microbiota’ (the detailed search format is provided in Appendix 1). The search time ranged from the establishment of the database to January 1, 2023 to retrieve all valuable animal models of SCI involving intestinal flora analysis; the search language and publication period were not limited (Figure 1).

### 2.2. Eligibility criteria

The inclusion criteria were as follows: (1) the type of research design was controlled, (2) the research object was animals, (3) the outcome indicators were intestinal flora abundance or indicators of the richness or diversity of intestinal flora, and (4) the original documents were published.

The exclusion criteria were as follows: (1) non-animal research object; (2) research not focused on the SCI model; (3) incomplete data, unable to extract the original data statistics literature; (4) conference papers, dissertations, and review articles; and (5) non-English papers.

### 2.3. Data extraction

All data were independently extracted from the original text or charts by two authors. (1) General characteristics: the first author, publication year, species of animals, age, weight, injured spinal section, and sampling time after SCI. (2) Outcome indicators study: alpha diversity (observed species index, Chao1 index, Shannon index, Simpson index, Pielou’s evenness, ACE index), bacterial phyla, bacterial orders, and bacterial genera.

Data was either directly extracted from the original text or emails were sent to the corresponding author of the study to request the original data. The GetData Graph Digitizer was used to obtain the relevant outcome indicator data if there was no response from the corresponding author or accurate original data could not be inability obtained [20].

### 2.4. Quality assessment

The risk of bias in the included studies was judged according to Cochrane Reviewer’s Handbook 5.1.0 [21] and was assessed by two independent authors using six criteria: (1) random sequence generation, (2) allocation concealment, (3) blinding of participants and personnel, (4) incomplete outcome data, (5) selective reporting, and (6) other biases. Each criterion was assessed as having low, unclear, or high bias. Two researchers independently scored the risk of bias and conducted cross-verification. Cases of disagreement involved asking a third party for advice after discussion. A random number table or generating random numbers by computer are regarded as low risk reliable methods for random sequence generation. Unreliable methods such as artificially selecting numbers are regarded as high risk, and unknown generation methods are regarded as unclear. The risk for allocation concealment and blinding of participants and personnel depends on whether the blinding method is strictly and properly used. A blinding method that was likely to be damaged during the study was considered high risk; otherwise, it was considered low risk. Failure to report all pre-specified primary outcome measures was considered high-risk, whereas outcome measures in the fully published study plan were considered low-risk. Other biases depended on whether there was a significant risk of bias or falsification.

### 2.5. Data analysis

All the extracted data were converted into the same unit before calculations, and the continuous variables were expressed as the mean ± standard deviation according to the formula presented in Appendix 2 (m = median, a = the smallest value (minimum), b = the largest value (maximum)) [22]. Data analysis was performed using R 4.3.1. The results were expressed as mean differences with 95% confidence intervals (CIs). Chi-squared tests and I^2^ statistics were used to analyze heterogeneity. *p* < .1 and *I*^2^ > 50% indicated a high likelihood of heterogeneity in the data. A random-effects model was used owing to the anticipated heterogeneity. Moreover, a subgroup analysis was performed to better interpret the results of this study, if necessary. Statistical significance was set at *p* < .05.

## 3. Results

### 3.1. Search results

A total of 218 studies were retrieved up to January 1, 2023, after searching Web of Science, PubMed, and Embase. Two researchers screened the titles and abstracts of the references using EndNote to remove duplicates. A total of 114 references were excluded as they did not meet the inclusion criteria, and 104 potential references were included. Fifteen studies were included in our meta-analysis after two researchers read the full texts.

### 3.2. Characteristics of selected studies

The animal model of SCI was a rat or mouse in 14 studies and a pig in 1 study (Table 1). The T10 sections were selected by over half of the authors (10 studies) for the selection of the SCI sections, whereas the C5 sections were selected by Schmidt and EKA [34].

**Table 1.** General characteristics and outcome indicators in included studies.

No	Author/Year	Animal	Age	Weight	Spinal Injury Section	Sampling time after SCI	Outcome assessment			
1	Smith [23]	Male long Evans rats	NA	250–300 g	T10	28 d	**Alpha Diversity:** Observed species index Shannon Index	**Phylum:** F/B Ratio Bacteroidetes Verrucomicrobiota Firmicutes Proteobacteria	**Order:** Erysipelotrichales Verrucomicrobiales	**Genus:** Akkermansia Bacteroides
2	Cheng [24]	Female Sprague Dawley rats	2-3 months	200 ± 20 g	T11–T12	16 d	**Alpha Diversity:** Shannon Index Chao1 Index	**Phylum:** Bacteroidetes Actinobacteria Verrucomicrobiota Firmicutes Proteobacteria	**Order:** Clostridiales	**Genus:** Bacteroides Lactobacillus Alloprevotella
3	Doelman [25]	Female Yucatan pigs	NA	20-30 kg	T2 or T10	14 d	**Alpha Diversity:** Pielou’s evenness		**Phylum:** Bacteroidetes Actinobacteria Firmicutes Proteobacteria	
4	Du [26]	Female C57BL/6 mice	NA	NA	T4 or T10	21 d	**Alpha Diversity:** Shannon Index	**Phylum:** Bacteroidetes Actinobacteria Firmicutes Proteobacteria	**Genus:** Bacteroides Lactobacillus	
5	He [27]	Female C57BL/6 mice	8–12 weeks	20–25 g	T10	35 d	**Alpha Diversity:** Shannon Index	**Order:** Erysipelotrichales Verrucomicrobiales Bacteroidales Clostridiales	**Genus:** Akkermansia Lactobacillus Alistipes Alloprevotella Lachnospiraceae_ NK4A136_group	
6	Jing [28]	Female C57BL/6N mice	NA	18–22 g	T10	24 d	**Alpha Diversity:** Chao1 Index ACE Index		**Phylum:** Bacteroidetes Verrucomicrobiota Firmicutes Proteobacteria	
7	Kang [29]	Female C57BL/6J mice	6–8 weeks	18–22 g	T8–T10	14 d	**Alpha Diversity:** Observed species index Shannon Index Chao1 Index Simpson Index Pielou’s evenness		**Phylum:** F/B Ratio Bacteroidetes Actinobacteria Firmicutes Proteobacteria	
8	Kigerl [30]	Female C57BL/6 mice	NA	NA	T9	28 d	**Order:** Bacteroidales Clostridiales			
9	O’Connor [31]	Female Fischer rats	NA	NA	T10	32 d	**Alpha Diversity:** Observed species index Shannon Index			
10	Rong [32]	C57BL/6 mice	6 weeks	25 ± 2 g	T10	7 d	**Genus:** Akkermansia Bacteroides Lactobacillus Alloprevotella Lachnospiraceae_NK4A136_group			
11	Rong [33]	Female C57BL/6N mice	NA	18–22 g	T10	21 d	**Alpha Diversity:** Observed species index Shannon Index Chao1 Index Simpson Index ACE Index	**Phylum:** Bacteroidetes Actinobacteria Verrucomicrobiota Firmicutes Proteobacteria	**Genus:** Akkermansia Bacteroides Lactobacillus Alloprevotella Lachnospiraceae_ NK4A136_group	
12	Schmidt [34]	Female Lewis rats	NA	180-220 g.	C5	28 d	**Alpha Diversity:** Shannon Index			
13	Schmidt [35]	Female Lewis rats	8–9 weeks	180–220 g	C5	28 d	**Alpha Diversity:** Shannon Index		**Phylum:** F/B Ratio	
14	Myersa [36]	Female C57BL/6 mice	6–8 weeks	18–22 g	T9	42 d	**Phylum:** Bacteroidetes Firmicutes Proteobacteria			
15	Zhang [37]	Female C57BL/6 mice	NA	20–22 g	T10	28 d	**Alpha Diversity:** Shannon Index ACE Index			

Five researchers chose 28 d after the establishment of the SCI model as the most common fecal sample collection time. In fact, most studies obtained fecal samples within 28 d; Scott A. Myersa took the longest sampling time after modeling at 42 d.

There were primarily four outcome indexes: alpha diversity, phylum, order, and genus. The outcome indicators are shown in Table 1.

### 3.3. Risk of bias assessment

**Figure 2.**
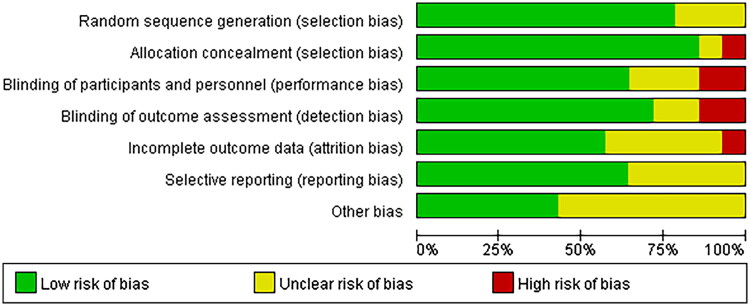
Overall quality of the included studies assessed by the Cochrane risk-of-bias assessment tool.

**Figure 3.**
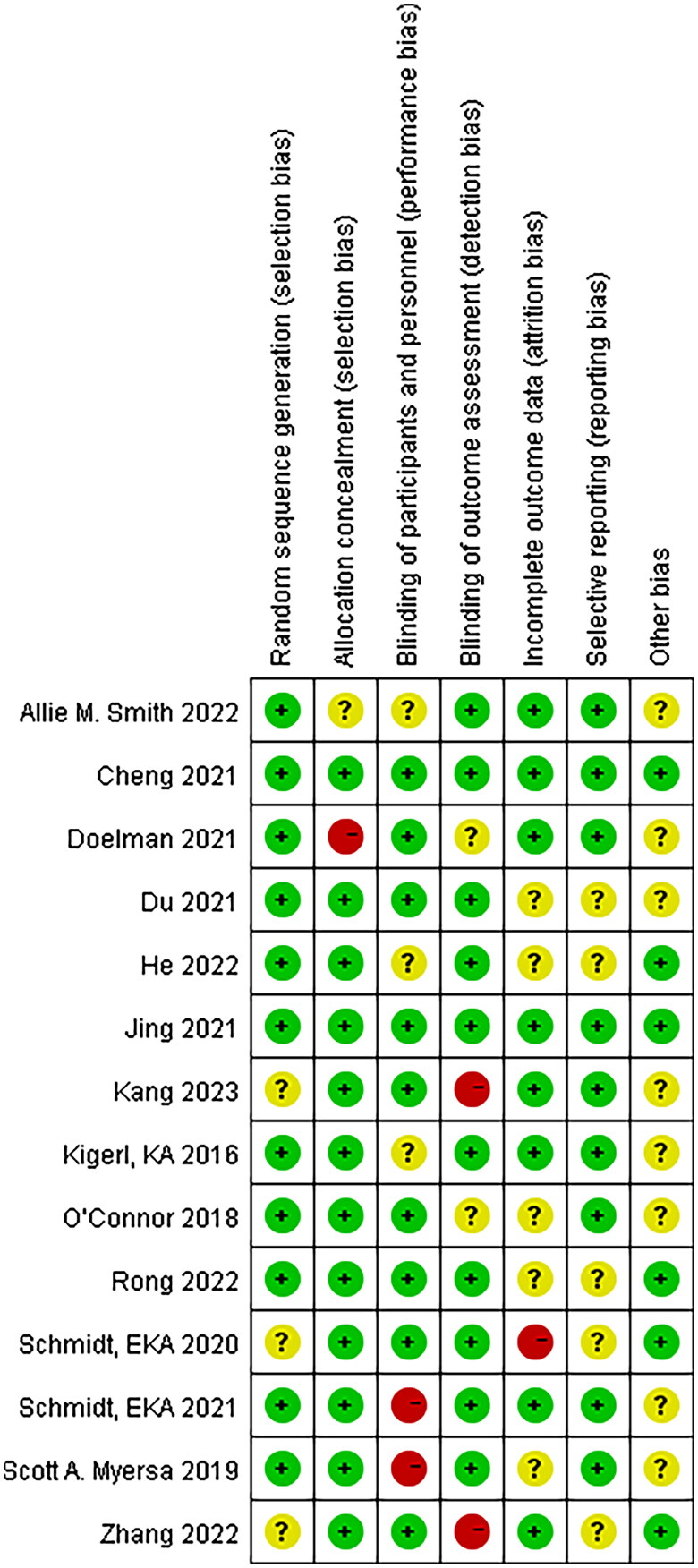
Risk of bias within studies assessed by the Cochrane risk-of-bias assessment tool.

All the studies were judged to have a low risk of bias, random sequences, allocation methods, and blinding. However, there is an unclear risk of bias owing to incomplete outcomes, data, and other biases (Figure 2 and 3).

### 3.4. Differences in outcome assessment between SCI models and the sham group

#### 3.4.1. Alpha diversity

**Figure 4.**
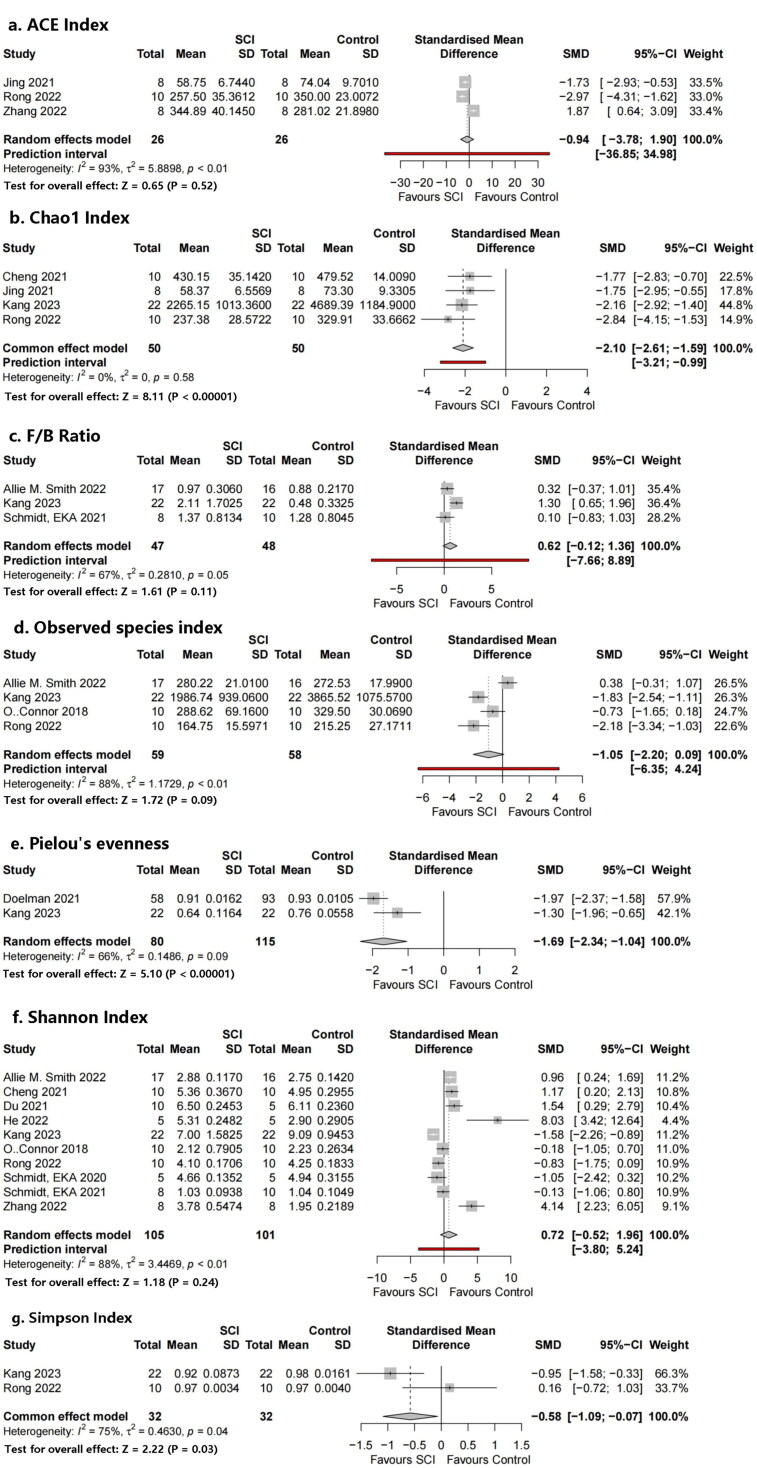
Forest plots of alpha diversity in the gut microbiota of the SCI group and the control group ((1) Observed species index, (2) Shannon index, (3) Chao1 index, (4) Simpson index, (5) Pielou’s evenness, (6) ACE index).

Several different types of alpha diversity indices were used to assess the microbial diversity within the same group, including estimated richness (observed species index, Chao1 index) and indices presenting richness and evenness (Shannon index, Simpson index, Pielou’s evenness, and ACE index) (Figure 4).

Alpha diversity is related to the number of species in a locally homogeneous habitat and is also known as intra-habitat diversity. β diversity (also known as between-habitat diversity) refers to the species composition of different habitat communities along environmental gradients or the rate of species replacement along environmental gradients. The Chao1 index was used to estimate the number of operational taxonomic units (OTUs) contained in a sample. The Chao1 algorithm is often used to estimate the total number of species in ecology, and is used to estimate the number of OTUs in a community. This was also proposed by Chao and is one of the common indices used to estimate the total number of species in ecology. Pielou’s evenness, Shannon index, and Simpson index were used to estimate the microbial diversity index in the samples and were often used to reflect the alpha diversity index. The higher the Shannon value, the higher the community diversity.

Four studies provided data on the observed species index and four studies provided data on the Chao1 index regarding richness. Subgroup analysis showed that the Chao1 indices in the SCI model group were significantly decreased compared with those in the sham group (standard mean difference (SMD) = −2.10, 95% CI −2.61 to −1.59, *p* < .00001, *I*^2^ = 0%;). The observed species index of the SCI model group was significantly lower than that of the control group in half of the four studies, although the meta-analysis did not show a statistically significant difference. There were no significant differences in SMDs of the observed species index (SMD = −1.05, 95% CI = −2.20 to 0.09, *p* = .09, *I*^2^ = 88%).

Ten studies provided data on Shannon’s index, two studies provided data on Simpson’s index, two studies provided data on Pielou’s evenness, and three studies provided data on the ACE index. Completely different results were observed in the 10 studies using Shannon’s index as the outcome indicator. Half of the studies found a significant increase in Shannon’s index in the SCI model group, whereas two studies showed significantly reduced Shannon’s indices; the remaining three studies found no statistically significant difference. No significant differences were found in our meta-analysis (SMD = 0.72, 95% CI = −0.52 to 1.96, *p* = .24, *I*^2^ = 88%). The number of articles included in the remaining three indicators was relatively small; Simpson’s indices in the SCI model group were significantly decreased compared with those in the control group (SMD= −0.58, 95% CI = −1.09 to −0.07, *p* = .03, *I*^2^ = 75%), and the same trend was found for Pielou’s evenness (SMD = −1.69, 95% CI = −2.34 to −1.04, *p* < .00001, *I*^2^ = 66%). Meanwhile, no significant differences were found in the ACE index (SMD = −0.94, 95% CI = −3.76 to 1.90, *p* = .52, *I*^2^ = 93%).

#### 3.4.2. Bacterial phyla

**Figure 5.**
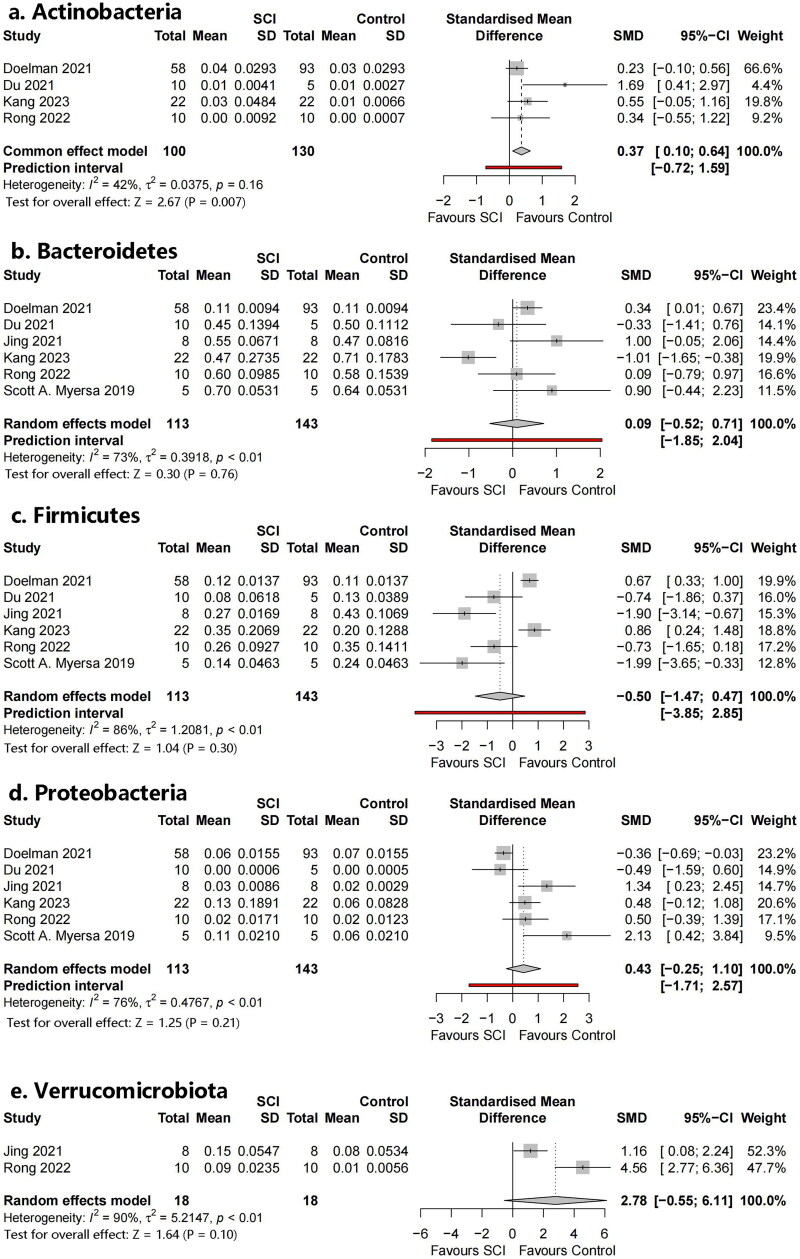
Forest plots of phyla in the gut microbiota of the SCI group and the control group ((1) Bacteroidetes, (2) Actinobacteria, (3) Verrucomicrobiota, (4) Firmicutes, (5) Proteobacteria, (6) Firmicutes/Bacteroidetes ratio (F/B ratio)).

Five phyla were identified (Figure 5): Bacteroidetes, Actinobacteria, Verrucomicrobia, Firmicutes, and Proteobacteria. Additionally, the Firmicutes/Bacteroidetes (F/B) ratio was included as an outcome index. Meta-analysis showed that Actinobacteria were statistically significant (SMD = 0.37, 95% CI 0.10–0.643, *p* = .007, *I*^2^ = 42%). There was no statistically significant difference in the level of the remaining phyla or the F/B ratio.

#### 3.4.3. Bacterial order

**Figure 6.**
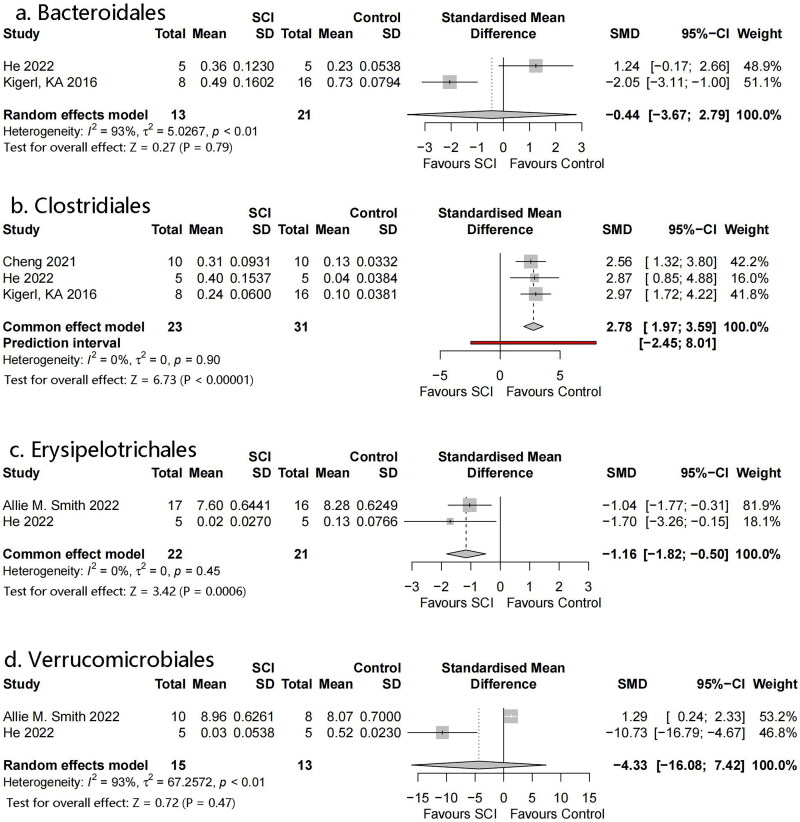
Forest plots of order in the gut microbiota of the SCI and control groups ((1) Erysipelotrichales, (2) Verrucomicrobiales, (3) Bacteroidales, (4) Clostridiales).

Four orders were identified: Erysipelotrichales, Verrucomicrobiales, Bacteroidales, and Clostridiales (Figure 6). Meta-analysis showed that Erysipelotrichales and Clostridiales had statistically significant differences: SMD = −1.16, 95% CI= −1.82 to −0.50, *p* = .0006, *I*^2^ = 0% and SMD = 2.78, 95% CI = 1.97–3.59, *p* < .00001, *I*^2^ = 0%, respectively. However, there were only a small number of studies included in both analyses. No significant differences were observed in the flora of the remaining orders.

#### 3.4.4. Bacterial genus

**Figure 7.**
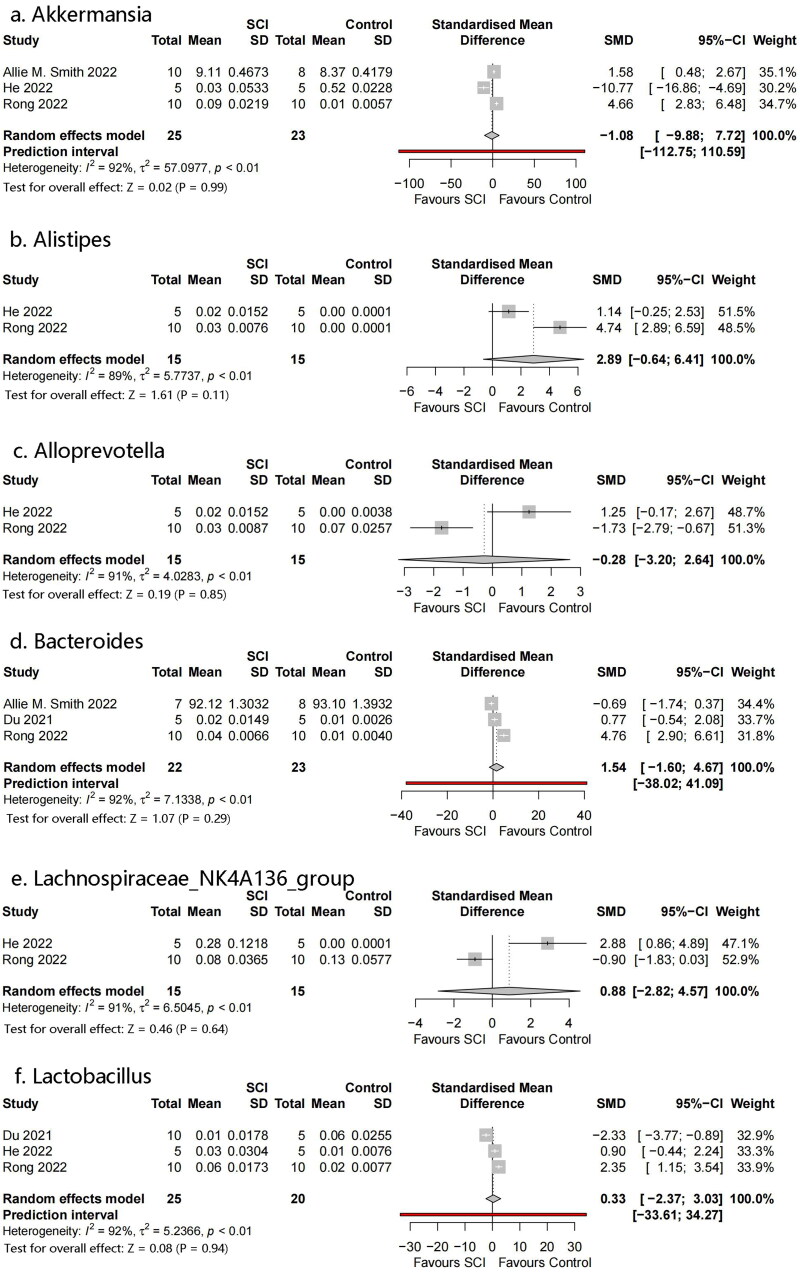
Forest plots of genera in the gut microbiota of the SCI and control groups ((1) Akkermansia, (2) Bacteroides, (3) Lactobacillus, (4) Alistipes, (5) Alloprevotella, (6) Lachnospiraceae_NK4A136_group).

Six genera were identified: *Akkermansia*, *Bacteroides*, *Lactobacillus*, *Alistipes*, *Alloprevotella*, and *Lachnospiraceae NK4A136* (Figure 7). No significant differences were observed between the genera.

**Figure 8.**
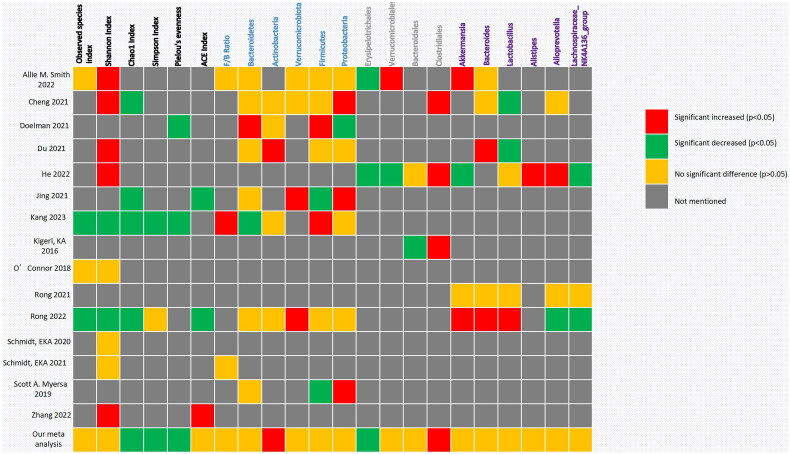
Summary outcomes of the included studies.

Figure 8 summarized the profile of the microbiota included in this study, including alpha diversity and relative abundance at the phylum, species, and genus levels. Different studies produced variable, or even completely opposite results. Consistent experimental results were observed for some indicators of alpha diversity. For example, all four included studies reported that the Chao1 index significantly decreased after SCI. Otherwise, some indicator experimental results were completely opposite to those reported by other researchers. For example, Shannon’s index (a commonly used indicator measuring bacterial richness) significantly increased in five studies after SCI, whereas two researchers concluded that it significantly decreased. This was especially true for the phylum flora. Meta-analysis showed statistically significant increases in Actinobacteria, Firmicutes, and Bacteroidetes (which have attracted much attention). Furthermore, there were often significantly different results owing to differences in experimental methods, such as different spinal cord sections or sampling times.

**Figure 9.**
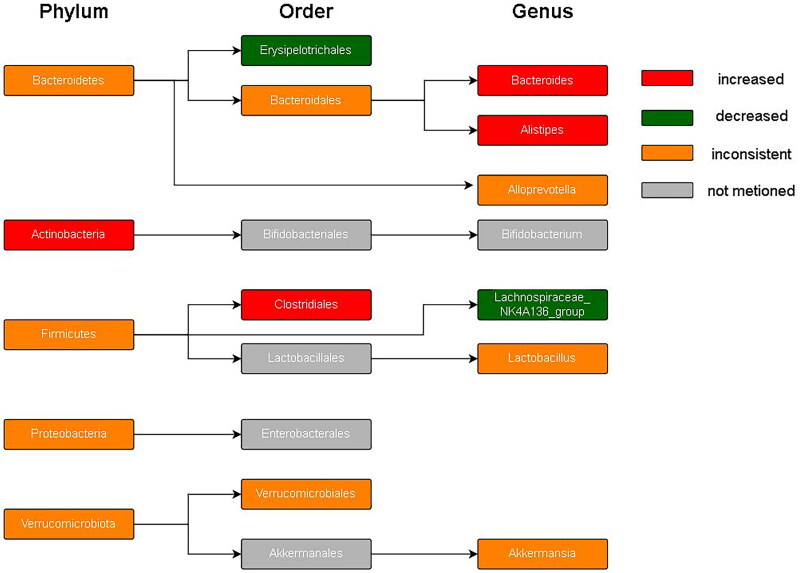
Hierarchical clustering.

Figure 9 shows the phylogenetic characteristics of rich taxon differences in mice with SCI compared to those seen in normal mice at the phylum, order, and genus levels, and the colors indicate different bacterial variations. Red, green, orange and grey indicate increased abundance, decreased abundance, inconsistent variation and abundance not mentioned, respectively.

## 4. Discussion

Many studies have systematically analyzed existing studies on intestinal flora and SCI; however, no meta-analysis had been performed. To the best of our knowledge, this is the first meta-analysis investigating the relationship between changes in gut microbiota and SCI in animal models. We searched three of the most commonly used databases for determining the changes in the diversity, richness, and evenness of the gut microbiota after SC, and the possible characteristics of bacteria that preserve spinal cord function from impairment.

Fifteen high-quality studies were included in this meta-analysis. The following indicators were included to describe the overall characteristics of the gut microbiota according to previous studies on intestinal flora: observed species index: Shannon’s index, Chao1 index, Simpson’s index, ACE index, and Pielou’s evenness [38,39]. In our analysis, three of the six alpha diversity measures were statistically significant. Among them, the Chao1 and Simpson indices (representing richness) and Pielou’s evenness significantly decreased, although studies of the latter two were limited. Shannon’s index is the most common indicator of microbiota diversity; however, different researchers have obtained varied or opposite results. Five of the 10 studies involving Shannon’s index reported a significant increase in SCI vs. healthy subjects and two reported a significant decrease.

Most studies included relevant experimental analyses regarding beta diversity. We did not conduct a meta-analysis owing to the insufficient number of studies with available data.

The characteristic gut microbiome taxa were explored by selecting bacteria from two or more studies with sufficient data for meta-analysis. Bacteroidetes and Firmicutes are the two most abundant phyla; this was not completely consistent with previous studies. Different, or even completely opposite conclusions were reached in different studies. We observed that Bacteroidetes showed an increasing trend; this correlated with Kang’s study [29]. Bacteroidetes are pro-inflammatory bacteria that can increase and aggravate symptoms after SCI. The dominant phylum Firmicutes in the intestinal tract was replaced by Bacteroidetes, the ratio of Firmicutes to Bacteroidetes decreased, and the abundance of Proteobacteria decreased. These bacterial translocations may be owing to increased permeability of intestinal epithelial cells when sympathetic nerves are inhibited and decreased gastrointestinal moisture and sensitivity of intestinal neurons to intestinal serotonin and nutrients when parasympathetic nerves are inhibited. This creates conditions for changes in the intestinal microenvironment [40,41]. The status of Firmicutes is complicated and research on this flora is currently unclear. Many people believe that it has a positive or side effect on SCI regulation, and we can obtain a glimpse of it from the heat map. The Firmicutes/Bacteroidetes ratio is considered an important indicator for describing intestinal disorders. However, no statistical significance was obtained in our analysis. This may be owing to the small sample size. Some studies suggest that the change in the ratio of Firmicutes to Bacteroides represented by a decrease in the abundance of Firmicutes and an increase in the abundance of Bacteroides is owing to a disorder of the intestinal microflora after SCI. Such changes in the microflora structure affect the production of SCFAs, resulting in severe inflammation and complex infections [42,43]. Actinobacteria has not received sufficient attention. In our analysis, Actinobacteria significantly increased in the SCI group. Its role in cellular and molecular mechanisms (such as bacterial translocation and inflammation induction) has not been clarified. The relative abundance of Clostridiales was significantly higher in SCI compared with healthy subjects, as described in many studies. No statistically significant bacterial genera were detected.

The close relationship between SCI and intestinal flora was reviewed by several researchers [44–49]. In this research, the influence of intestinal ecological changes after SCI is often mentioned. However, the relationship between intestinal flora changes and SCI is confusing [49]. Following SCI, inflammatory mediators are produced *via* numerous pathways [50]. Changes in gut microbiota can stimulate the production of cytokines such as IL-8, IL-10, and TNF in enteric-related lymphoid tissues that can reach the SCI site *via* the circulatory system and activate resting microglial cells in the central nervous system [51–53]. One type of microglia called M1 produces a various pro-inflammatory factors (such as TNF-α, IL-1β, IL-6, NO, and reactive oxygen species) that can induce the apoptosis of neurons and oligodendrocytes, cause secondary injury, aggravate the pathophysiological reaction of SCI, and affect the functional recovery of the injured spinal cord [54].

The gut microbiota-brain axis is an important topic in the study of intestinal flora and SCI. Experimental models of the central nervous system showed that the two-way communication between the central nervous system and the intestine was accompanied by ecological disorders after injury, neuroinflammatory responses mediated by gastrointestinal-associated lymphoid tissue, and neurotransmission of bacterial metabolites [55]. Studies suggest that the gut microbiota – brain axis is associated with psychosis. Emotional and psychological changes after SCI may be related to changes in the volume of specific brain regions involved in information processing and emotions that can produce anxiety- and depression-like symptoms, with an incidence between 11% and 30% [56].

At present, the study of intestinal flora has focused on pathogenesis. There are several proposed treatments for SCI, including FMT, melatonin therapy, and probiotic therapy [57,58]. Many treatments are being tested in animals and patients [34,57,59]. Both the direct transplantation of healthy feces and the implantation of specific probiotics regulate impaired immune function and improve intestinal barrier function by producing antibacterial products, such as organic acids [60]. An increasing number of studies were conducted on intestinal flora in the past 10 years, and breakthroughs and progress were made. However, the complex and specific composition of intestinal flora and the intestinal immune environment remind us that we should not ignore the differences between laboratory animals and humans, and between people of different races, genders, and ages [61]. Exploring valuable intestinal changes in a complex population with significant differences is a future focus for developing and improving the treatment of SCI.

This meta-analysis has some limitations. First, the animal models included in this study were not identical, including animal type, sex, weight, and age; therefore, it was impossible to accurately estimate their impact on outcome indicators. Second, the sampling time was not completely consistent (although most were within 28 d or less), The longest sampling time was 42 d, while the shortest was 7 d However, the times were still within the traditional perception of acute SCI. We initially intended to perform subgroup analyses of features from different studies (such as the type of subjects studied and the timing of fecal sampling) or the SCI section to better illustrate the results. However, it was difficult to obtain meaningful subgroup analysis results from the chaotic data from different studies; therefore, we did not conduct a subgroup analysis. The inability to accurately analyze the effects of acute and chronic SCI on gut microbiota changes is an unresolved problem [49]. Undeniably, there is no clear evidence to determine whether a phase effect causes significant changes in the intestinal flora within this period. Third, the small number of studies, the insufficient sample size of each study, and the high heterogeneity of some included studies resulted in limited statistical power.

## 5. Conclusion

Our meta-analysis suggested that the gut microbiota in the animal model group with SCI showed varying degrees of changes in richness and evenness compared to that in the control group, and potentially harmful characteristic flora were identified. More rigorous methodological studies are needed owing to the high heterogeneity and limited sample size. Further research is needed to clinically apply the intestinal microbiota and possibly guide FMT therapy.

## Data Availability

Data supporting the results of this study can be obtained through the corresponding author. 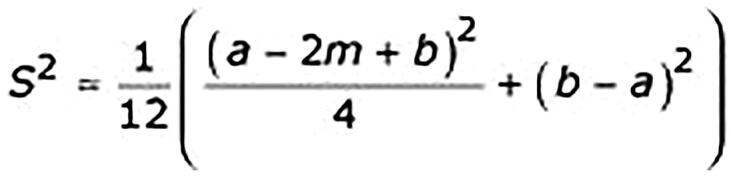

## Appendix

### 1. Search strategy for PubMed

**Table:**

ID	Search	Hits
#1	(((((intestinal microbiota[MeSH Terms]) OR (gut microbiota[MeSH Terms])) OR (Intestinal immunity[MeSH Terms])) OR (gut immunity[MeSH Terms])) OR (Microbiota[MeSH Terms])) OR (Microbiome[MeSH Terms])	91,102
#2	(((((intestinal microbiota[Title/Abstract]) OR (gut microbiota[Title/Abstract])) OR (Intestinal immunity[Title/Abstract])) OR (gut immunity[Title/Abstract])) OR (Microbiota[Title/Abstract])) OR (Microbiome[Title/Abstract])	118,518
#3	#1 OR #2	153,006
#4	((((spinal cord injury[Title/Abstract]) OR (SCI[Title/Abstract])) OR (spinal cord trauma[Title/Abstract])) OR (acute spinal injury[Title/Abstract])) OR (chronic spinal injury[Title/Abstract])	65,236
#5	(spinal cord injury[MeSH Terms]) OR (spinal cord trauma[MeSH Terms])	55,503
#6	#4 OR #5	89,387
#7	#3 AND #6	124
**Final Run Date: January 1, 2023**		

## 2.

### Search strategy for EMBASE

**Table:**

ID	Search	Hits
#1	‘intestinal microbiota’/exp	95,686
#2	'intestinal microbiota’:ti,ab,kw OR ‘gut microbiota’:ti,ab,kw OR ‘intestinal immunity’:ti,ab,kw OR ‘gut immunity’:ti,ab,kw OR ‘microbiota’:ti,ab,kw OR ‘microbiome’:ti,ab,kw	143,757
#3	#1 OR #2	173,353
#4	'spinal cord injury’/exp	93,208
#5	'spinal cord injury’:ab,ti OR ‘sci’:ab,ti OR ‘spinal cord trauma’:ab,ti OR ‘acute spinal injury’:ab,ti OR ‘chronic spinal injury’:ab,ti	88,017
#6	#4 OR #5	131,054
#7	#3 AND #6	268
**Final Run Date: January 1, 2023**		

**3. Search strategy for Web of Science**

**Table:**

ID	Search	Hits
#1	(((((TS=(intestinal microbiota)) OR TS=(gut microbiota)) OR TS=(Intestinal immunity)) OR TS=(gut immunity)) OR TS=(Microbiota)) OR TS=(Microbiome)	238,853
#2	TS=(intestinal microbiota* OR gut microbiota* OR Intestinal immunity* OR gut immunity* OR Microbiota* Microbiome*)	160,419
#3	#1 OR #2	238,897
#4	TS=(‘spinal cord injury’ OR ‘SCI’ OR ‘spinal cord trauma’ OR ‘acute spinal injury’ OR ‘chronic spinal injury’)	189,101
#5	TS=(pain$)	1,815,030
#6	#4 OR #5	1,992,423
#7	#3 AND #6	3,409
**Final Run Date: January 1, 2023**		
